# Recombinant Production of Snakin-2 (an Antimicrobial Peptide from Tomato) in *E. coli* and Analysis of Its Bioactivity

**DOI:** 10.3390/molecules200814889

**Published:** 2015-08-14

**Authors:** Vera Herbel, Holger Schäfer, Michael Wink

**Affiliations:** Institute of Pharmacy and Molecular Biotechnology (IPMB), Heidelberg University, Im Neuenheimer Feld 364, D-69120 Heidelberg, Germany; E-Mails: Herbel@uni-heidelberg.de (V.H.); Holger.schaefer@uni-heidelberg.de (H.S.)

**Keywords:** snakin-2, antimicrobial peptide, *Escherichia coli*, recombinant expression

## Abstract

Antimicrobial peptides (AMPs) represent a diverse group of biologically active molecules that are part of the innate immune systems of a variety of organisms. Their primary function consists of protecting the host organism against invading microorganisms, including pathogens. AMPs show a broad spectrum of secondary structures, which are essential for antimicrobial activity. In this study, we produced snakin-2 (SN2), a 66-amino-acid-(aa)-long AMP from *Solanum lycopersicum* as a recombinant protein in *E. coli*. This AMP belongs to the GASA/GAST protein family and possesses a highly conserved 60-aa-long domain with six disulfide bonds in the *C*-terminus of the peptide. Because of the toxicity of SN2 against its producing *E. coli* strain, the AMP was attached to an *N*-terminal fusion protein (thioredoxin A), which was removed after affinity chromatography purification. The total yield of recombinant SN2 was approximately 1 mg/L. The membrane-active SN2 showed a bactericidal and fungicidal bioactivity, which can be explained by perforation of biomembranes of bacteria and fungi.

## 1. Introduction

Pathogens like bacteria, fungi, viruses, or parasites cause a wide range of critical diseases in humans, animals, and plants. Furthermore, increasing numbers of multidrug-resistant microorganisms, which are able to withstand common antimicrobial drugs, represent a serious threat. Therefore, it is necessary to search for new types of antimicrobial agents that are nontoxic for the infected organism but active against multidrug-resistant strains. Antimicrobial peptides (AMPs) represent an interesting group of bioactive and antimicrobial natural products. They are small peptides, typically consisting of 12–60 or more amino acids. AMPs are an ancient evolutionary part of the innate immune systems of all complex living organisms [1,2]. Host defense is the primary role of AMPs, and they target a wide spectrum of pathogenic microorganisms like bacteria and fungi as well as viruses [2].

Although the antimicrobial effect is the same within different groups of AMPs, structural similarities are less evident due to highly diverse amino acid sequences. Roughly, AMPs can be categorized in four structural groups: AMPs containing amphipathic α-helices, two to four β-strands, loop structures, and extended structures. The mode of action of antibacterial peptides is based on the interaction of the cationic AMP with the negatively charged phospholipids in the bacterial cell membrane [3]. There are four models proposed, including the formation of membrane pores lined with AMPs in a “barrel-stave” or “torroid” manner, or spanning the membrane as an “aggregate” of peptides and lipids [4,5,6,7]. 

In plants, a wide variety of AMPs are present in all tissues and can be constitutively expressed or induced upon infection [8]. Plant AMPs share some characteristics such as small molecular weight (2–10 kDa), cationic net charge, and 2–6 disulfide bonds, which stabilize the whole peptide. Their activity is directed against pathogenic microorganisms such as bacteria, fungi, or oomycetes [9]. 

The snakin peptide family is characterized by maintaining 12 cysteins in the highly conserved *C*-terminus of the peptide, which form six disulfide bridges in the mature protein. These bonds are not only important for the secondary structure of the peptide [10,11] but are also essential for its biological activity [12]. Two snakin peptides from potato (StSN1 and StSN2) were further characterized by overexpression or isolation from potato tubers, and it was shown that both of those peptides mediate strong antimicrobial activity against plant pathogens [13,14,15]. Overexpression of the tomato snakin-2 (SN2) enhanced the tolerance of transgenic tomato plants against *Clavibacter michiganensis* [16]. By silencing the snakin-2 gene in tobacco plants (*Nicotiana benthamiana*), susceptibility to *Clavibacter michiganensis* was enhanced [17].

In our study, we produced the SN2 peptide from tomato (*Solanum lycopersicum*) for the first time as a recombinant peptide in *E. coli*. This is possible by its joint expression as a fusion protein with the fusion partner thioredoxin (Trx), which is present on the expression vector pET-32c(+). For obtaining a high amount of SN2, the parameters for its recombinant expression were optimized. The fusion partner was enzymatically removed by the TEV protease cleavage after protein purification. Antibacterial and antifungal activities of the recombinant SN2 were characterized.

## 2. Results and Discussion

### 2.1. Construction of the pET-32c(+)-SN2 Expression Vector

Antimicrobial peptides are limited in clinical applications because of complicated extraction procedures from their natural source or their cost-intensive chemical synthesis. For large-scale production of AMPs, the recombinant DNA technique is used to obtain bioactive but inexpensive peptides [18]. *E. coli* is an easy-to-handle and fast-growing expression host that can be used for expression of recombinant AMPs originating in many organisms, including the tomato [19,20]. In addition, it is recommended to express an AMP with a non-toxic fusion partner such as maltose-binding protein or thioredoxin (Trx) to increase the solubility and to avoid toxicity for the host cells [19,21].

**Figure 1:**
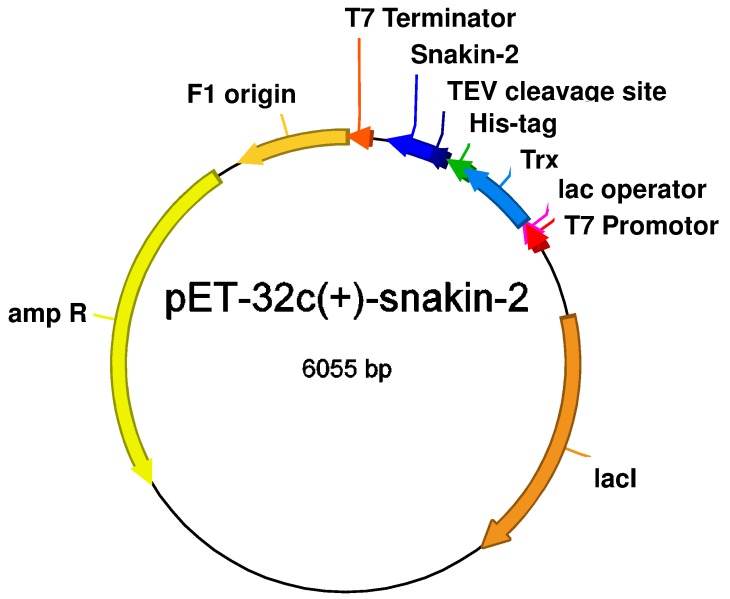
Schematic representation of the pET-32c(+)-SN2 expression vector.

In our study, the recombinant expression vector pET-32c(+)-SN2 was prepared by Eurofins Genomics (Ebersberg, Germany). The codon-optimized SN2 gene was fused to a Trx-tag to mask the antimicrobial activity during the expression in *E. coli*. The coding sequence for a His-tag is present on the pET-32c(+) vector between the SN2 and Trx genes, and the coding sequence for a TEV protease cutting site was cloned to the 5′ end of the SN2 gene. The expression of the whole fusion protein is regulated by the T7 promotor to minimize spontaneous expression (Figure 1).

### 2.2. Expression, Purification, and Proteolytic Cleavage of Trx-SN2

The parameters necessary for the highest yield of the soluble Trx-SN2 fusion protein expressed in *E. coli* BL21[DE3] were determined via SDS-PAGE; conditions include expression at 37 °C for 2 h after induction with 0.7 mM IPTG (isopropyl β-d-1-thiogalactopyranoside). However, it has been reported that lower expression temperatures could increase the solubility of the recombinant protein [22].

**Figure 2:**
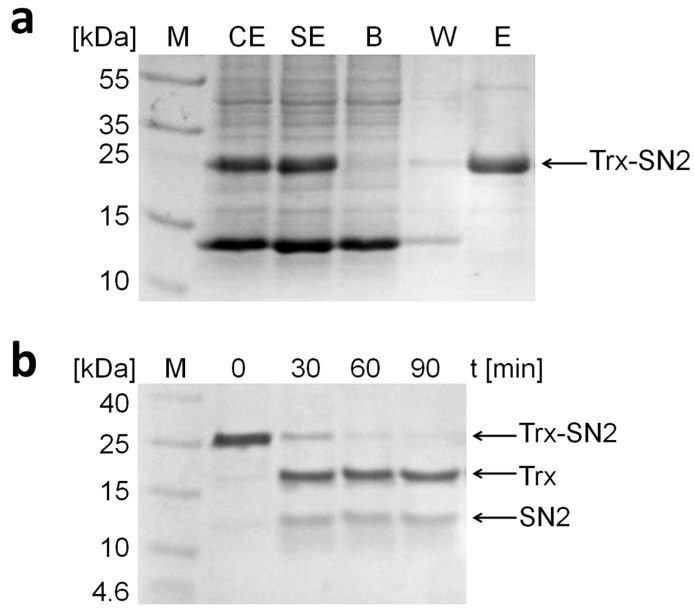
SDS-PAGE analysis (12%) of the purification steps of the Trx-SN2 fusion protein. (**a**) CE is the crude and SE is the soluble protein extract derived from the *E. coli* lysate. B represents the binding step in which the fusion protein is bound to the column; W shows the washing step; and E the final elution step where the purified fusion protein is released from the column. M is the protein molecular weight marker (Fermentas, Germany); (**b**) SDS-PAGE analysis (12%) of the cleaving activity of the TEV protease on the Trx-SN2 fusion protein. After 90 min, the fusion protein is cut into two parts. The band at 17.6 kDa represents Trx and the one appearing at 12.5 kDa represents SN2. For possible explanations of the aberrant migration behavior of SN2 (Mr = 7.1 kDa), see text.

After preparation of the soluble protein extract (SE), the fusion protein was purified by affinity chromatography (Figure 2a). The fusion protein was bound to a Ni-TED column (binding step, B) and eluted by adding 250 mM imidazole to the buffer (elution step, E). The fusion protein has a molecular weight of 24.7 kDa (determined by the ExPASy Compute pI/Mw tool, Swiss Institute of Bioinformatics, http://www.expasy.org/).

The fusion protein was later cleaved by the TEV protease because additional protein tags can interfere with the biological activity of the recombinant peptide [23]. Several cleavage durations were tested (Figure 2b), and after 90 min, the whole fusion protein was cut into two parts which can be identified on a SDS-PAGE as bands with a molecular weight of 17.6 kDa (Trx) and 12.5 kDa (SN2). This tag removal can cohere with a loss of recombinant protein when the cleavage is inefficient. However, in our case, the TEV protease cuts up to 95% of the fusion protein within 90 min, and incubation overnight does not result in any unspecific cleavage. The yield of the Trx-SN2 fusion protein is roughly 3 mg/L *E. coli* culture and purified recombinant SN2 is approximately 1 mg/L bacterial culture.

The band of the snakin-2 peptide seems to be larger than the expected 7.1 kDa on the SDS-PAGE. This anomalous migration behavior during electrophoresis can have different reasons [24]. Here, we assume that the cationic nature of the peptide does not allow for the SDS molecules to mask all the positive charges within the peptide. This leads to a slower migration rate and the peptide seems larger on the SDS-PAGE than it actually is.

We tried to separate the recombinant snakin-2 from the thioredoxin with different methods (affinity chromatography, ion exchange chromatography), but these attempts were not successful. Thus, we decided to work with the mixture of these two proteins. Thioredoxin was additionally expressed and used as a negative control in every experiment investigating bioactivity. Thioredoxin showed no effect, so we conclude that the measured bioactivity is caused by snakin-2.

**Figure 3:**
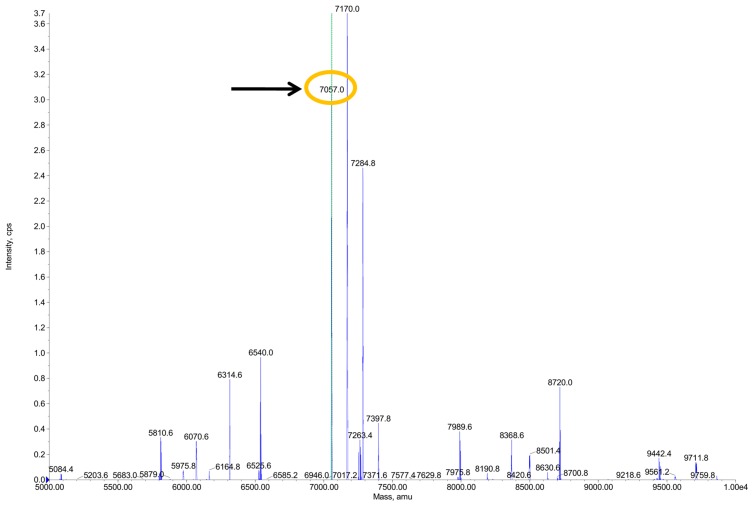
HPLC-ESI-QTOF analysis of SN2. The expected mass of 7057 Da was confirmed and gave evidence that all six disulfide bonds are present in the mature peptide.

HPLC-ESI-QTOF analysis of SN2 confirmed the expected mass of 7057 Da (Figure 3). The theoretically calculated mass of the SN2 peptide was 7069 Da (determined by the ExPASy Compute pI/Mw tool), but due to the loss of 12 hydrogen atoms by forming six disulfide bonds, the mature peptide possesses a molecular weight of 7057 Da. This result confirms that the recombinant peptide exhibits the right amino acid composition. Furthermore, it demonstrates the formation of all six disulfide bonds in the mature protein and shows that the recombinant SN2 was not denaturated during the purification procedure.

The mass spectrum shows that beside the peak at 7057 Da (which is snakin-2), there are two other peaks with the masses of 7170 and 7284.8 Da. During the electrospray ionization process, peptides can be ionized with a different number of TFA (trifluoroacetic acid) molecules. The peaks with masses of 7170 and 7284.8 Da are also snakin-2 peptides, but possess an adduct of TFA, which is added in the HPLC to mask positive charges of the peptides. TFA has a mass of 113 Da, so the peak at 7170 Da is a snakin-2 peptide with one additional TFA molecule and the peak at 7284.8 Da represents a snakin-2 peptide with two additional TFA molecules. In addition, the HPLC chromatogram shows the exact same elution time for the three peptides with the masses of 7057, 7170, and 7284.8 Da. Different species of peptides would have different elution time points.

### 2.3. Analysis of the SN2 Bioactivity

#### 2.3.1. Perforation of the Biomembrane

**Figure 4:**
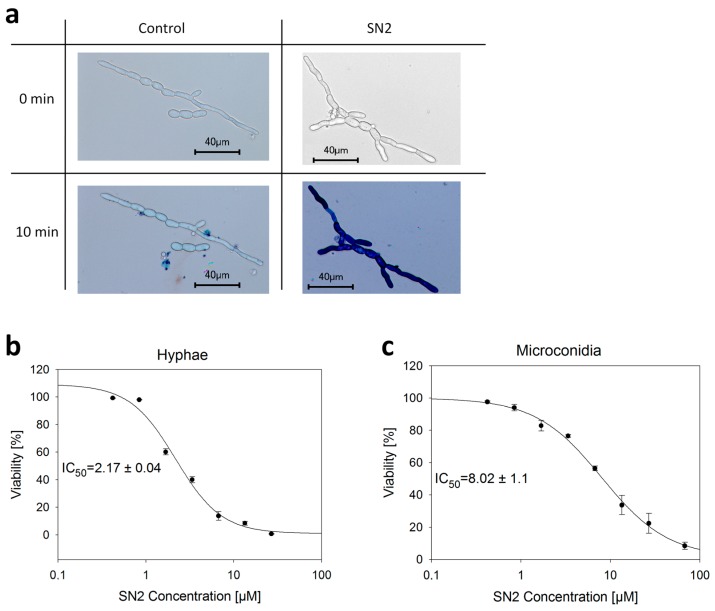
Trypan blue assay. (**a**) Perforating effect of SN2 on the biomembrane of *F. solani*. The control shows no stained cells whereas SN2-treated cells are stained dark blue after the addition of the trypan blue dye. Pictures were taken with the microscope software (Keyence BZ-9000 Viewer); (**b**,**c**) Analysis of the viability of hyphae and microconidia of *F. solani* by performing the trypan blue assay with several concentrations of SN2. IC_50_ values were determined using a four-parametric logistic curve (Sigma Plot 11.0).

Trypan blue is a diazo dye commonly used for testing cell viability because it can only infiltrate cells with a perforated biomembrane, whereas healthy cells will not be stained [25]. In our study, the trypan blue assay was used to give evidence that SN2 is perforating biomembranes of microorganisms. Therefore, an SN2 solution was applied on hyphae and microconidia cells from *Fusarium solani* (a pathogenic mold from Solanaceae), and after the addition of trypan blue (0.5%), the reaction was observed under the microscope (Figure 4a). It is clearly visible that SN2-treated cells are stained blue compared to the control, which indicates that SN2 is perforating the biomembrane of the hyphal cells, allowing the dye to penetrate the SN2-treated cells. A similar conclusion was reached for microconidia.

To study the viability of hyphae and microconidia cells of *F. solani*, SN2 was serially diluted for the trypan blue assay. The viability was quantified by calculating the percentage of stained and unstained cells, respectively. SN2 exhibits an IC_50_ value of 2.17 ± 0.04 µM for hyphae and 8.02 ± 1.1 µM for microconidia (Figure 4b). These values cannot be easily compared to literature data because the concentration of cells used for this assay is four times higher than is suggested by EUCAST (The European Committee on Antimicrobial Susceptibility Testing) (1 × 10^5^ cfu/mL) [26]. To count stained and unstained cells, the number of cells is higher in this assay to ensure the presence of approximately 1000 cells per microscope slide. We used the microdilution assay to compare our results to literature values.

In many instances, we could observe under the microscope that after the application of the snakin solution and trypan blue dye, the fungal membrane broke open, the trypan dye infiltrated the cell at specific points, and, with time, the complete cell was stained dark blue. This effect was not found in the negative control, so we hypothesized that snakin-2 can perforate biomembranes. In the future, more detailed studies will be performed.

#### 2.3.2. Antimicrobial Activity of SN2

The antibacterial and fungicidal effect was determined using the microdilution assay. MIC (minimum inhibitory concentration), MMC (minimum microbicidal concentration), and IC_50_ (half maximal inhibitory concentration) values are documented in Table 1. SN2 mediates strong microbicidal activity against all tested strains (gram-negative bacteria, gram-positive bacteria, and fungi) with MIC values between 0.26 and 8.49 µM. IC_50_ values for SN2 in the tested bacterial strains are all lower than 1 µM, which is also correct for the yeast *P. pastoris*. Only the mold *F. solani* shows a slightly higher IC_50_ value of 1.58 µM for SN2.

**Table 1:** Antimicrobial activity of recombinant SN2.

Organism		MIC (µM)	MMC (µM)	IC_50_ (µM)
*E. coli*	gram^−^	4.25	8.49	0.9 ± 0.3
*A. tumefaciens*	gram^−^	1.06	Not tested	0.41 ± 0.13
*M. luteus*	gram^+^	0.26	1.06	0.11 ± 0.03
*S. cohnii*	gram^+^	1.06	2.12	0.44 ± 0.1
*P. pastoris*	yeast	8.49	16.99	0.91 ± 0.06
*F. solani*	mould	4.25	8.49	1.58 ± 0.24

These values are comparable to other AMPs such as ranalexin, hydramacin-1, and Ib-AMP4 [27,28,29]. Our results are also in good agreement with data from other snakin peptides from potato (StSN1 and StSN2) or pepper (CaSn) [14,15,30]. The SN2 peptide does not distinguish between bacterial or fungal cell membranes. For both kinds of microorganisms, we found a strong antimicrobial effect, which presumably originates from perforating the biomembrane (Figure 4). According to these results, SN2 is apparently a representative of a new class of peptide antibiotics.

#### 2.3.3. Agglomerating Effect of SN2

**Figure 5:**
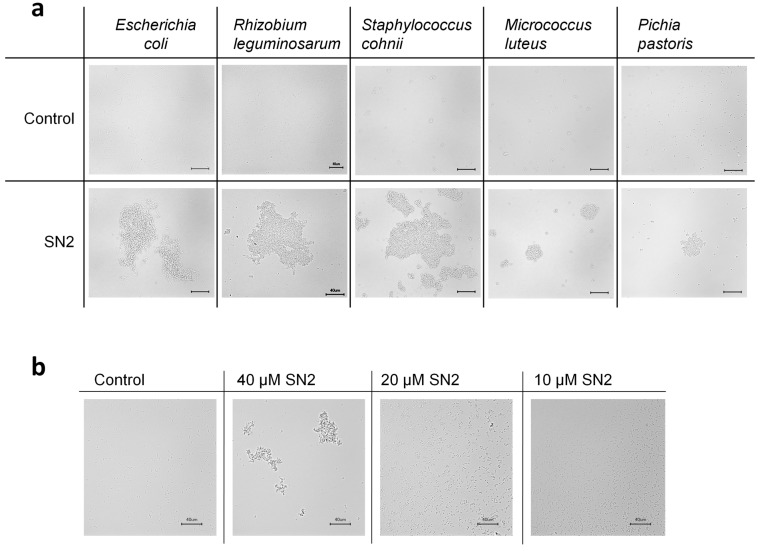
(**a**) Aggregation of bacteria and yeast after the addition of SN2; (**b**) dose-dependence of the agglomerating effect on *E. coli* caused by SN2. The scale bar represents 40 µm.

A rapid agglomerating effect of SN2 was found for all tested microorganisms (Figure 5a). This effect was described before for the snakin peptides StSN1 and StSN2 from potato [14,15]. It was supposed that this effect could prevent the diffusion of the pathogens outside of the wounded plant area into uninfected zones. Also, hydramacin-1 from the metazoan hydra causes the aggregation of bacterial cells [28].

Furthermore, our study investigated that this agglomerating effect is dose-dependent, which means that the size of agglomerates decreases proportionally with the concentration of SN2. The agglomerating effect could be based on the cationic nature of the peptides, which may function as a bridge between the negatively charged bacterial membranes. For investigating the practical use, we are currently performing further experiments.

## 3. Experimental Section

### 3.1. Strains, Plasmids, Enzymes, and Media

*E. coli* strain BL21[DE3] was used as expression host in combination with the pET-32c(+) expression plasmid (Novagen, Germany). The TEV protease and DNaseI were purchased from Sigma-Aldrich GmbH (Munich, Germany), IPTG and lysozyme from AppliChem GmbH (Darmstadt, Germany). For culturing *E. coli* and expression of the fusion protein, Luria-Bertani (LB) Broth (1% tryptone, 0.5% yeast extract, 1% NaCl) was used. In the antimicrobial tests, salt-free LB (1% tryptone, 0.5% yeast extract) was used for bacteria and Sabouraud-Glucose (SAB) Broth (2% mycological peptone, 4% D(+)-glucose) for fungi. To prepare solid media 1.5% agar was added. The bioactivity of SN2 was analyzed against gram-negative bacterial strains (*E. coli* DH5α, *Agrobacterium tumefaciens*), gram-positive strains (*Micrococcus luteus*, *Staphylococcus cohnii*), and fungi (*Pichia pastoris*, *Fusarium solani*).

### 3.2. Cloning of the SN2 Gene into the pET-32c(+) Expression Vector

The DNA sequence of the SN2 gene was codon-optimized for the expression in *E. coli*. The sequence coding for a TEV protease cutting site was attached at the 5′-end of the gene and two stop codons (TAA, TGA) were added at the 3′-end of the gene to terminate the translation. The gene was synthesized and cloned into the pET-32c(+) vector between the restriction sites of *Kpn*I and *Xho*I by Eurofins Genomics (Ebersberg, Germany). In the recombinant vector pET-32c(+)-SN2, the SN2 gene was fused to a Trx-tag and a His-tag, both of which were present in the original vector.

The recombinant expression vector was transformed into chemically competent *E. coli* BL21[DE3] cells, and after PCR colony screening, the DNA sequences of the positive clones were confirmed by DNA sequencing.

### 3.3. Expression and Purification of Trx-SN2

For the expression, a single colony was inoculated in LB (5 mL, 100 µg/mL ampicillin). This preparatory culture was used to inoculate the expression culture (300 mL LB, 100 µg/mL ampicillin) which was incubated at 37 °C with shaking at 220 rpm. As soon as the optical density (OD) of the cells, measured at 600 nm, reached a value of 0.6, the expression of the fusion protein was induced by addition of IPTG. Several incubation temperatures, expression durations, and IPTG concentrations were tested and analyzed by SDS-PAGE. The highest yield of soluble fusion protein was obtained at 37 °C for 2 h after induction with 0.7 mM IPTG.

After expression, the cells were harvested by centrifugation (10 min, 3000× *g*, 4 °C) and a soluble protein extract was prepared by resuspending the cell pellet in 5 mL/g pellet LEW (lysis-wash-equilibration) buffer (Macherey-Nagel, Düren, Germany). After addition of lysozyme (1 mg/mL) and DNaseI (5 µg/mL), the cells were lysed during incubation on ice for 45 min, with shaking at 300 rpm. After pelleting the cell debris (30 min, 10,000× *g*, 4 °C), the soluble protein extract can be used for purification by affinity chromatography. Therefore, the Protino^®^ Ni-TED columns (Macherey-Nagel, Düren, Germany) were used. According to the manufacturer’s instructions, the fusion protein was purified, and the purification steps were analyzed via SDS-PAGE. 

### 3.4. Proteolytic Cleavage of Trx-SN2

Proteolytic cleavage was performed in the elution buffer of the affinity purification (50 mM NaH_2_PO_4_, 300 mM NaCl, 250 mM imidazole, pH 8.0) with addition of EDTA (ethylenediaminetetraacetic acid, 10 mM) and TEV protease (protease-to-target protein ratio (*w*/*w*) of 1:100) for 2 h at 30 °C. The following purification of SN2 from the Trx was not successful, so the mixture of SN2 and Trx was used for experiments on bioactivity. Trx alone was expressed from the pET-32c(+) vector after inserting a stop codon at the 3′-end of the Trx gene using mutagenesis (Q5^®^ Site-Directed Mutagenesis Kit, NEB, Frankfurt am Main, Germany). This Trx protein was used as a control in all experiments relating to bioactivity.

After cleavage, the buffer was exchanged for water by size exclusion chromatography using PD10 desalting columns (GE Healthcare, Solingen, Germany) according to the manufacturer’s instructions. The protein concentration was determined by the Bradford assay described previously [31]. In case of a low protein concentration, the peptide solution was concentrated using Amicon ultra centrifugal filters (Merck KGaA, Darmstadt, Germany). As a final step, the peptide solution was sterile filtrated (Minisart, Sartorius, Göttingen, Germany).

### 3.5. Trypan Blue Assay

The viability of fungal hyphae and spores (microconidia) was assessed by the trypan blue assay. The microconidia of *F. solani* were separated from the hyphal cells by filtration (Schleicher & Schuell filter paper named 595½, 90 mm, GE Healthcare, Solingen, Germany).

According to the EUCAST, a final cell concentration of 1 × 10^5^ cfu/mL for molds should be used [26]. For this assay, a concentration of 2 × 10^5^ cfu/mL was used to ensure that sufficient cells are available under the microscope to be counted. Then, 5 µL of serially diluted SN2 solution was mixed with 5 µL of cells (4 × 10^5^ cfu/mL) and incubated for 10 min at 28 °C. Immediately after adding 2 µL of 0.5% trypan blue, stained and unstained cells were counted under the microscope (Keyence BZ-9000, KEYENCE Deutschland GmbH, Neu-Isenburg, Germany) and the percentage of viability was calculated using Sigma Plot 11.0 (four-parametric logistic curve, Systat Software, Inc., San Jose, CA, USA, www.sigmaplot.com). Recombinant Trx was used as control.

### 3.6. Microdilution

The microdilution method was used to determine the minimal inhibitory concentration (MIC) and minimal microbicidal concentration (MMC) values. Recombinant SN2 was serially diluted in water to obtain concentrations between 35–0.01 µM (240–0.1 µg/mL) in 96-well plates. After twofold dilution with salt-free LB (bacteria) or SAB (fungi) medium, microbial suspensions of 1 × 10^6^ cfu/mL (bacteria) or 1 × 10^5^ cfu/mL (fungi) were added, and the plates were incubated at 37 °C (*E. coli*, *M. luteus*, *S. cohnii*) or 28 °C (*A. tumefaciens*, *P. pastoris*, *F. solani*) for 24 h. The first concentration at which no turbidity could be observed was determined as MIC. To define the MMC value, 3 µL of every clear well were inoculated on solid media and incubated for 24 h at 37 °C or 28 °C. The concentration at which no visible growth arose was defined as MMC. The IC_50_ values were defined as 50% reduction in cell viability and were calculated using Sigma Plot 11.0 (four-parametric logistic curve). Recombinant Trx was used as control.

### 3.7. Agglomeration Studies

For agglomeration, 5 µL of a bacterial (1 × 10^6^ cfu/mL) or yeast cell (1 × 10^5^ cfu/mL) suspension was mixed with 5 µL of SN2 (40 µM), and immediately a photograph was taken under the microscope (Keyence BZ-9000, KEYENCE Deutschland GmbH, Neu-Isenburg, Germany). Recombinant Trx was used as control.

## 4. Conclusions

Snakin-2 (SN2) was successfully expressed in *E. coli* as a bioactive recombinant peptide. To mask the antimicrobial activity, the peptide was produced as a fusion protein with an *N*-terminal Trx-tag. After affinity chromatography purification, the fusion partner was enzymatically removed, and the molecular weight and formation of the six disulfide bonds were confirmed by HPLC-ESI-QTOF analysis. The mature peptide showed strong microbicidal activity against bacteria and fungi, mediated through perforation of the biomembrane. Furthermore, it led to a dose-dependent agglomeration of cells, which is supposed to diminish the distribution of pathogens inside the plant.
